# Analysis of Functional Promoter of Camel FGF21 Gene and Identification of Small Compounds Targeting FGF21 Protein

**DOI:** 10.3390/vetsci10070452

**Published:** 2023-07-10

**Authors:** Fang Yong, Meilin Yan, Lili Zhang, Wangye Ji, Shuqin Zhao, Yuan Gao

**Affiliations:** 1 College of Life Science and Technology, Gansu Agricultural University, Lanzhou 730070, Chinazhaosq@gsau.edu.cn (S.Z.); 2 Gansu Key Laboratory of Animal Generational Physiology and Reproductive Regulation, Lanzhou 730070, China

**Keywords:** camel FGF21, promoter, bioinformatics, molecular docking

## Abstract

**Simple Summary:**

The fibroblast growth factor 21 (FGF21) gene plays an important role in the mechanism of glucose and lipid metabolism and is a promising therapeutic target for metabolic disease. Camel displayed a unique regulation characteristic of glucose and lipid metabolism, endowing them with the ability to adapt to survive under drought and chronic hunger. However, the knowledge about the camel FGF21 gene regulation and its differences between humans and mice is still limited. This study obtained the camel FGF21 promoter sequence, determined its core active regions and specific regulatory pattern different from humans and mice, and further screened two potential small molecules targeting FGF21 protein using molecular docking and in silico ADMET druggability prediction. This study expanded the functions of these small molecules and provided a foundation for drug development targeting FGF21.

**Abstract:**

The fibroblast growth factor 21 (FGF21) gene plays an important role in the mechanism of glucose and lipid metabolism and is a promising therapeutic target for metabolic disease. Camels display a unique regulation characteristic of glucose and lipid metabolism, endowing them with the ability to adapt to survive drought and chronic hunger. However, the knowledge about the camel FGF21 gene regulation and its differences between humans and mice is still limited. In this study, camel FGF21 gene promoter was obtained for ~2000 bp upstream of the transcriptional start site (TSS). Bioinformatics analysis showed that the proximal promoter region sequences near the TSS between humans and camels have high similarity. Two potential core active regions are located in the −445–612 bp region. In addition, camel FGF21 promoter contains three CpG islands (CGIs), located in the −435~−1168 bp regions, significantly more and longer than in humans and mice. The transcription factor binding prediction showed that most transcription factors, including major functional transcription factors, are the same in different species although the binding site positions in the promoter are different. These results indicated that the signaling pathways involved in FGF21 gene transcription regulation are conservative in mammals. Truncated fragments recombinant vectors and luciferase reporter assay determined that camel FGF21 core promoter is located within the 800 bp region upstream of the TSS and an enhancer may exist between the −1000 and −2000 bp region. Combining molecular docking and in silico ADMET druggability prediction, two compounds were screened as the most promising candidate drugs specifically targeting FGF21. This study expanded the functions of these small molecules and provided a foundation for drug development targeting FGF21.

## 1. Introduction

Fibroblast growth factor 21 (FGF21) is an atypical member of the FGF family and has no mitogen activity in vivo, but is characterized by a lower binding affinity for heparin, which enables it to be transported in the circulatory system and function in an endocrine manner and act as effective regulators of glucose and lipid metabolism [1,2,3]. Camel FGF21 protein, with high homology with mice and humans, is a polypeptide containing 209 amino acid residues. The camel FGF21 coding gene is located on chromosome 9. FGF21 is a hormone that regulates glucose–lipid metabolic pathways, including stimulating the oxidation of fatty acids, production of ketone bodies, inhibition of lipogenesis, glucose uptake, amino acid transport, and energy expenditure [4,5,6]. Thus, FGF21 is involved in many metabolic diseases, such as obesity, diabetes, and non-alcoholic fatty liver [7,8,9], making it a promising therapeutic target for these metabolic diseases. Actually, FGF21 analogs LY2405319, PF-05231023, etc., were proven to be effective therapeutic agents in obesity, type 2 diabetes mellitus (T2DM), non-alcohol fatty liver disease (NAFLD) and cholestatic liver disease [10,11]. Camel displayed a unique regulation characteristic of glucose and lipid metabolism, endowing them with the ability to adapt to survive under drought and chronic hunger [12]. However, the knowledge about camel FGF21 gene regulation and its differences between humans and mice is still limited.

Promoters and enhancers are two classes of activating regulatory elements that drive important gene transcription processes. Promoter defines where transcription is initiated and enhancers are elements that amplify such transcription initiation [13]. Transcription factors bind to the elements in promoters or enhancers and form a pre-initiation complex with RNA polymerase, priming RNA polymerase for transcription. Promoters lie directly upstream of transcription start sites (TSS) so TSS are central to the identification of core promoters [14,15]. The disorders in promoter regulation directly affect gene expression and are associated with some diseases [16]. Much research on transcriptional regulation has been described recently, such as finding genomic patterns associated with promoter activity and using bioinformatics to predict candidate active promoters or enhancers based on genomic patterns. The promoter reporter assay is the most common method in promoter screening and research [16,17].

Molecular docking, an established in silico structure-based method with the ability to identify novel compounds of therapeutic interest, predict ligand-target interactions at a molecular level, and delineate structure-activity relationships without knowing a priori the chemical structure of other target modulators, has become an important common component of the drug discovery toolbox [18,19]. This helps to shorten the cycle of new drug development, reduce the cost of drug development, and speed up the drug development process. In this study, camel FGF21 gene promoter was obtained for bioinformatics analysis of active regions, CpG islands and transcription factor binding prediction. The camel FGF21 core promoter region was determined by truncated fragments recombinant vectors and luciferase reporter assay. In addition, molecular docking and in silico absorption, distribution, metabolism, and excretion (ADMET) prediction were performed to screen potential small binding molecules. This study attempted to provide a basis for studying the specific regulation of the camel FGF21 gene and for drug research targeting the FGF21 protein.

## 2. Materials and Methods

### 2.1. Promoter Cloning and Construction

Camel, human and mouse *FGF21* gene potential promoter sequences were analyzed and obtained for ~2000 bp upstream of TSS of Bactrian camel, human and mouse *FGF21* gene with gene ID: 105062602, 26291, 56636 in GenBank database, respectively. Bactrian camel tissue was obtained as mentioned previously [20]. Genomic DNA from camel kidney tissue was extracted according to the Genomic DNA Extraction Kit Novizan Biotechnology (Nanjing, China). Full length and walking truncated camel promoters were cloned and inserted into pGL4.10 plasmid to generate a luciferase report recombinant vector. The primers used for promoter cloning and constructions were shown as follows. Camel FGF21-promoter-2099F: 5′-ACCTCGAGTGAAGGAGATCTTGGTGGAT-3′. Camel FGF21-promoter-1087F: 5′-ACCTCGAGTCCCCGGACCGGGAGATCC-3′. Camel FGF21-promoter-831F: 5′-ACCTCGAGACCTGGATGCGCCGAGTCT-3′. Camel FGF21-promoter-678F: 5′-ACCTCGAGTCCACCCACAAATCAGAAC-3′. Camel FGF21-promoter-469F: 5′-ACCTCGAGTCTGCAGACCTGAAGCGCT-3′. Camel FGF21-promoter-262F: 5′-ACCTCGAGGTCCAATCTCTTGCACCCT-3′. Camel FGF21-promoter-R: 5′-GCAAAGCTTACCCAAACACCAAACATC-3′. Restriction sites are underlined. The primers were synthesized by GenScript (Nanjing, China). The PCR product and all recombinant vectors were verified by DNA sequencing by Sangon (Shanghai, China).

### 2.2. Cell Culture and Luciferase Reporter Assay

HEK293T cells were cultured in Dulbecco’s modified Eagle’s medium (DMEM) containing 10% fetal bovine serum (FBS) in a 5% CO_2_ incubator at 37 °C. Before transfection, cells were seeded into a 24-well plate and cultured for 70–80% confluence; transfection was performed with Lipofectamine 2000 reagent (Life Technologies, Carlsbad, CA, USA) according to the manufacturer’s protocols. pRL-TK (Promega, Madison, WI, USA), as an internal reference luciferase reporter, was co-transfected with pGL4.10-promotor recombinant vectors. Cells were harvested at 36 h post-transfection. Firefly and Renilla luciferase activities were determined using a dual-luciferase reporter assay system (Promega) according to the manufacturer’s instructions. Data represent relative firefly luciferase activity normalized to Renilla luciferase activity. All experiments were repeated at least three times.

### 2.3. Bioinformatics Analysis of Promoters

Promoter sequence analysis was performed using bioinformatics methods. Briefly, sequence alignment was performed by DNAMAN and BLAST (https://blast.ncbi.nlm.nih.gov/Blast.cgi, accessed on 13 July 2022). Prediction of cytosine-guanine (CpG) islands (CGIs) was performed using MethPrimer (http://www.urogene.org/methprimer, accessed on 11 September 2022). The promoter core active region was analyzed by Neural Network Promoter Prediction (https://fruitfly.org/seq_tools/promoter.html, accessed on 11 September 2022). Analysis of transcription factor binding site was performed using PROMO (https://alggen.lsi.upc.edu/recerca/menu-recerca.html, accessed on 11 September 2022), Match-1.0 Public (http://gene-regulation.com/pub/programs.html, accessed on 11 September 2022) and Cister (https://bu.wenglab.org/cister/cister.shtml, accessed on 11 September 2022).

### 2.4. Binding Analysis of Small Compounds Targeting FGF21 Protein

Potential FGF21 targeting small molecule compounds and the potential drug binding pocket were predicted and analyzed using CavityPlus online platform (http://repharma.pku.edu.cn/cavityplus, accessed on 21 March 2023). UCSF DOCK6 program (https://dock.compbio.ucsf.edu/DOCK_6/index.htm, accessed on 21 March 2023) was conducted to screen potential ligands binding to FGF21 protein from over 2700 small molecule compounds in DrugBank molecular library (https://go.drugbank.com, accessed on 21 March 2023) based on the FGF21 protein structure virtually. The binding modes of 2D and 3D between FGF21 protein and small compounds were displayed using PyMol and LigPlot software (Version 2.2).

### 2.5. In Silico ADMET Druggability Prediction

SwissADME (http://www.swissadme.ch/, accessed on 23 June 2023) network software was used to predict the druggability of the top five small molecule drugs with the binding force. Potential medicinal properties in the ADMET model were evaluated from the following six aspects: physical and chemical properties, fat solubility, water solubility, Pharmacokinetic properties, drug-like properties, and medicinal chemistry properties. The prediction results were digitized and potential medicinal small molecules were identified under Lipinski’s rule of five [21]: the number of hydrogen bond donors ≤ 5, the number of hydrogen bond acceptors ≤ 10, the octanol-water partition coefficient log P ≤ 5, the molecular mass ≤ 500 Da, and the number of rotatable bonds ≤ 10.

## 3. Results

### 3.1. Alignment of FGF21 Promoter Sequence

**Figure 1:**
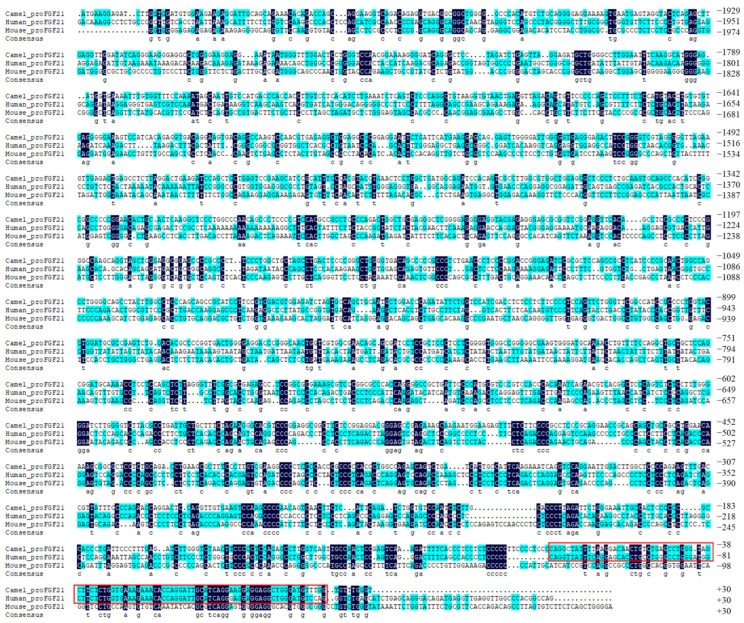
Alignment of camel, human and mouse FGF21 promoter sequences. Dark blue indicates the same nucleotide in all three sequences. Cyan color indicates the nucleotides that are identical in two sequences. The red boxes indicate areas with high sequence similarity.

FGF21 is a key hormone that regulates glucose–lipid metabolism and energy expenditure [4,5]. To investigate the regulation of the FGF21 gene at the transcription level and understand the differences among camel, human and mouse promoter sequences of ~2000 bp upstream of the transcription initiation site of the FGF21 gene were obtained from the GenBank database and used for alignment in this study. As shown in Figure 1, there are significant differences in FGF21 promoter sequences among different species, although mammalian FGF21 protein is highly conserved [22], indicating that significant differences in transcriptional regulation of the FGF21 gene exist among different species. Intriguingly, the proximal promoter region sequences near the TSS between humans and camels have high similarity (Figure 1 red box), suggesting that some conservative regulatory models exist in this region.

### 3.2. Analysis of Core Active Region of FGF21 Promoter

To understand the transcriptional activity of camel FGF21 promoter and screen the core active region, the Neural Network Promoter Prediction network tool was used to analyze camel, mouse and human FGF21 promoter sequences. As shown in Table 1, two potential core active regions with a score > 0.8 were found in the camel FGF21 promoter, which is located in the −445~−495 bp and the −562~−612 bp region, respectively. In addition, two potential core active regions were also found in the mouse FGF21 promoter sequence with the localization of the −18~−68 bp and the −2049~−2099 bp region, respectively. No highly active regions were found in the human promoter. The result indicated that some important regulatory sites may exist at the −445~−612 bp upstream of camel FGF21 gene TSS.

### 3.3. CGIs Prediction of FGF21 Promoter Sequence

**Figure 2:**
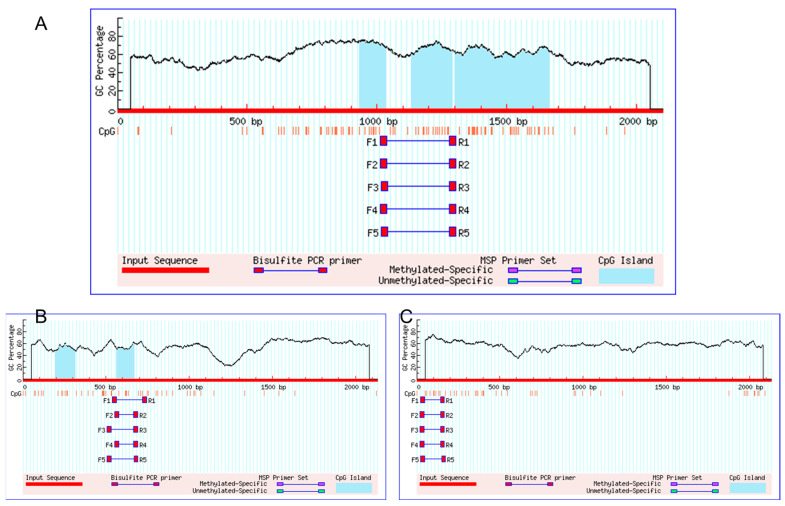
CGIs prediction of camel, human and mouse FGF21 promoter sequence. (**A**): camel. (**B**): human. (**C**): mouse.

CGIs are generically equipped to influence local chromatin structure and simplify the regulation of gene activity [23]. To further explore the transcriptional activity regulation of the camel FGF21 gene, this study performed CGIs prediction using MethPrimer online software (Version 2.0). As shown in Figure 2, there were three potential CGIs in camel FGF21 promoter, which were located in the −435~−800 bp, −811~−969 bp and −1067~−1168 bp regions, respectively (Figure 2A). In contrast, only two short CGIs were found in human FGF21 gene distal promoter region of −1568~−1460 bp and −1936~−1817 bp, respectively (Figure 2B) and no potential CGIs were found in mouse FGF21 promoter (Figure 2C). The result indicated that camel FGF21 gene transcription may be more active.

### 3.4. Analysis of Potential Transcription Factors Binding to FGF21 Promoter

**Figure 3:**
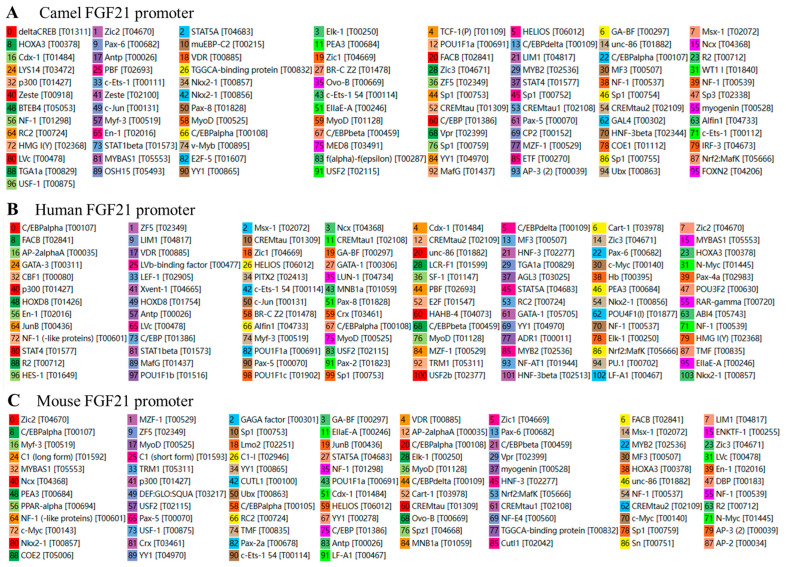
Putative main transcription factor binding site in the FGF21 promoter region.

To investigate the potential transcription factor binding sites in FGF21 promoter, several network software including PROMO, Match-1.0 Public and Cister was used to predict and analyze potential transcription factor binding sites in the promoter sequences of camel, human and mouse *FGF21* genes. As shown in Figure 3 and Tables S1–S3, collectively, 96, 103, and 91 potential transcription factor binding sites were found in the FGF21 promoter region of camel, human and mouse, respectively. Most transcription factors found are the same in different species although the binding site positions in the promoter are different. In addition, this study further investigated the binding of some major functional transcription factors such as SP1, NF-1, GATA1, YY1 and Elk-1 on promoters and found that these transcription factors nearly appear in all mammals (Table 2, Tables S1 and S3). The result indicated that the signaling pathways involved in FGF21 gene transcription regulation are conservative in mammals.

### 3.5. Determination of the Core Active Region of Camel FGF21 Promoter

**Figure 4:**
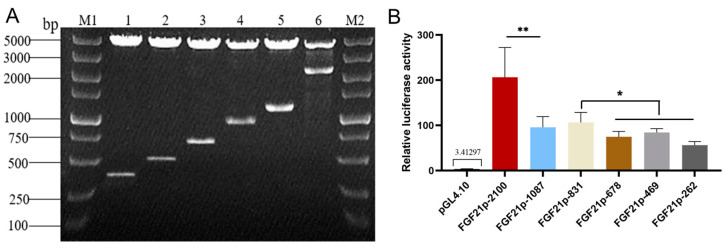
Construction of camel FGF21 walking truncated promoter reporter vectors and determination of the core active region of camel FGF21 promoter. (**A**): Enzyme digestion analysis of camel FGF21 walking truncated promoter reporter vectors. M1, M2: DNA Marker. (**B**): Determination of the core active region of camel FGF21 promoter using luciferase reporter assay. * *p* < 0.05, ** *p* < 0.01 (Student’s *t*-test).

To determine the core active region of camel FGF21 promoter, camel FGF21 promoter with 2100 bp upstream of TSS was cloned from camel tissue genome and inserted into luciferase reporter vector, pGL4.10. Walking truncated fragments with an interval of ~200 bp were generated using primer amplification and inserted into pGL4.10 to construct a series of promoter reporters. The luciferase reporter assay was performed to evaluate the promoter fragment activity. As shown in Figure 4, six camel FGF21 promoter fragments recombinant vectors were constructed successfully, termed as pGL-FGF21p-2100, pGL-FGF21p-1087, pGL-FGF21p-831, pGL-FGF21p-678, pGL-FGF21p-469 and pGL-FGF21p-262, respectively (Figure 4A). The results of the luciferase report found that all six recombinant reporter plasmids showed significantly higher relative luciferase activity than pGL4.10 empty plasmid (Figure 4B), indicating that the fragments upstream of TSS have strong promoter activity. In addition, pGL-FGF21p-2100 showed significantly higher luciferase activity than pGL-FGF21p-1087 and other shorter fragments, suggesting that some important functional elements that activate transcription maybe exist in the distal promoter region. In the proximal promoter region, pGL-FGF21p-831 showed the highest luciferase activity, indicating that the core promoter of camel FGF21 is located in the 800 bp region upstream of the TSS, and an enhancer may exist between the −1000 and −2000 bp region.

### 3.6. Analysis of Potential Drug-Binding Pocket of Camel FGF21 Protein

**Figure 5:**
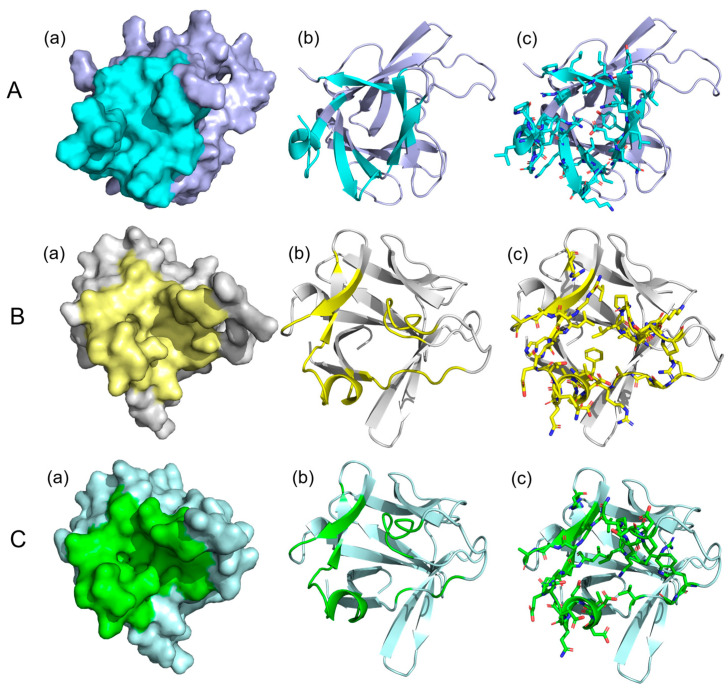
FGF21 protein drug binding pocket surface diagram. (**A**): The surface (**a**), backbone conformation (**b**), and spatial structure (**c**) of camel FGF21 protein drug binding pocket. (**B**): The surface (**a**), backbone conformation (**b**), and spatial structure (**c**) of human FGF21 protein drug binding pocket. (**C**): The surface (**a**), backbone conformation (**b**), and spatial structure (**c**) of mouse FGF21 protein drug binding pocket. The potential drug binding sites were colored in darker in FGF21 protein.

The finding that camel FGF21 regulates glucose–lipid metabolism has made it a promising therapeutic target for metabolic disease [20]. To study the potential binding sites of small molecular drugs on FGF21 protein, the potential drug-binding pocket of FGF21 protein was analyzed using the CavityPlus online platform. As shown in Figure 5, the secondary structure of camel FGF21 protein contains multiple β-strands and an α-helix, forming potential a drug-binding pocket (Figure 5A). The pocket represented in cyan and is composed of 36 discontinuous amino acid residues, including Leu-165, Ile-91, Gly-41, Gln-43, Phe-123, Pro-166, Pro-88, Tyr-132, Ala-85, Gly-131, Leu-38, Gln-124, Gly-42, Leu-127, Gln-39, Glu-125, Leu-126, Lys-87, Leu-86, Pro-36, Arg-45, Arg-47, Leu-128, Leu-167, Gly-89, Asn-133, Phe-164, Leu-83, Lys-84, Pro-168, Phe-40, Gln-92, Val-90, Tyr-135, Val-44, Leu-37. Human and mouse FGF21 proteins have drug-binding pockets with similar spatial structure (Figure 5B,C), but with varying amino acid residues forming the pockets compared to camel FGF21 protein (Table S4). The result indicated that FGF21 showed structural conservatism in mammals.

### 3.7. Screening of Potential Small Compounds Targeting FGF21 Protein

**Figure 6:**
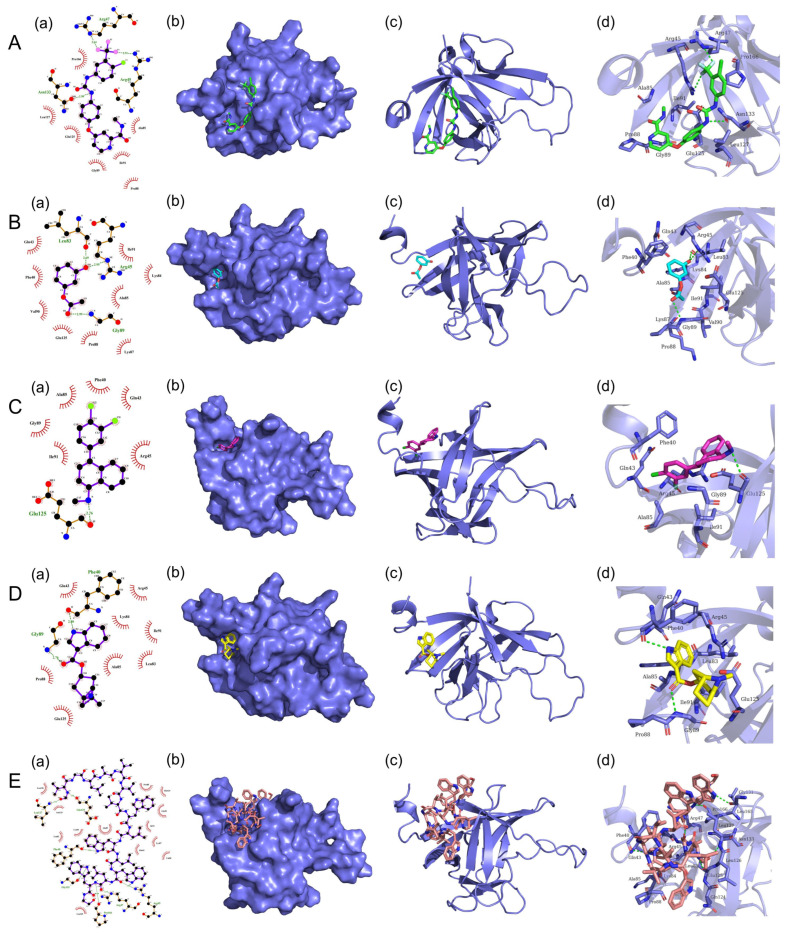
Binding pattern of small molecule compounds with FGF21 protein. (**A**): The 2D (**a**), surface (**b**) and 3D (**c**,**d**) binding model of Sorafenib and FGF21. Sorafenib was colored green. (**B**): The 2D (**a**), surface (**b**) and 3D (**c**,**d**) binding model of Resorcinol monoacetate and FGF21. Resorcinol monoacetate was colored in cyan. (**C**): The 2D (**a**), surface (**b**) and 3D (**c**,**d**) binding model of Sertraline and FGF21. Sertraline was colored pink. (**D**): The 2D (**a**), surface (**b**) and 3D (**c**,**d**) binding model of Tropisetron and FGF21. Tropisetron was colored yellow. (**E**): The 2D (**a**), surface (**b**) and 3D (**c**,**d**) binding model of Gramicidin D and FGF21. Gramicidin D was colored orange.

To further screen the potential small compounds targeting FGF21 protein, over 2700 small molecule compounds from DrugBank molecular library were performed for high throughput docking against FGF21 protein using the UCSF DOCK6 program. The first 1000 small molecules with binding ability to camel FGF21 protein were shown in Table S5, and the top five potential drug molecules with strong binding forces were selected for analysis. Their physical and chemical properties binding to FGF21 are shown in Table 3, and their 2D and 3D binding modes were analyzed by using PyMol and LigPlot software. As shown in Table 3 and Figure 6, the small molecule, Sorafenib, showed the strongest binding force against the FGF21 protein. Two fluorine atoms of Sorafenib act as hydrogen bond acceptors to form hydrogen bonds with Arg47 and Arg45 residues in FGF21, and one nitrogen atom of Sorafenib acts as a hydrogen bond donor to form hydrogen bonds with Asn133 residues in FGF21. In addition, Sorafenib forms hydrophobic interactions with FGF21 residues including Pro166, Leu127, Glu125 (Figure 6A). The interaction between Resorcinol monoacetate and FGF21 protein is shown in Figure 6B. An oxygen atom of Resorcinol monoacetate acts as a hydrogen bond donor and acceptor, forming two hydrogen bonds with the Leu83 and Arg45 residues in FGF21, respectively. The other oxygen atom of Resorcinol monoacetate acts as a hydrogen bond acceptor and forms a hydrogen bond with the Gly89 residue in FGF21. Additionally, Resorcinol monoacetate forms hydrophobic interactions with FGF21residues including Gln43, Phe40, Val90, and Glu125 (Figure 6B). The interaction between Sertraline and FGF21 protein is shown in Figure 6C. A nitrogen atom of Sertraline acts as a hydrogen bond donor and forms a hydrogen bond with the Glu125 residue in the FGF21 protein. Additionally, Sertraline forms hydrophobic interactions with FGF21 residues including Gly89, Ile91, Ala85, and Phe40 (Figure 6C). The interaction between Tropisetron and FGF21 protein is shown in Figure 6D. A nitrogen atom of Tropisetron acts as a hydrogen bond donor and forms a hydrogen bond with the Phe40 residue in FGF21. An oxygen atom of Tropisetron acts as a hydrogen bond acceptor and forms a hydrogen bond with the Gly89 residue in FGF21. Additionally, Tropisetron forms hydrophobic interactions with FGF21 residues including Gln43, Arg45, Ile91 and Lys84 (Figure 6D). The interaction between Gramicidin D and FGF21 is shown in Figure 6E. Four nitrogen atoms of Gramicidin D act as hydrogen bond donors to form hydrogen bonds with FGF21 residues Glu125, Phe40, Gly131 and Pro166, respectively. Three oxygen atoms of Gramicidin D act as hydrogen bond acceptors and form hydrogen bonds with FGF21 residues Leu127, Arg47 and Arg45, respectively. Additionally, Gramicidin D forms hydrophobic interactions with FGF21 residues Leu126, Asn133, Leu83, Lys84, and Ala 85 (Figure 6E). The results provide a basis for further screening, analysis, and validation.

Effective and safe drugs exhibit a finely tuned combination of pharmacodynamics and pharmacokinetics, including high potency, affinity and selectivity against the molecular target, along with adequate absorption, distribution, metabolism, excretion and tolerable toxicity (ADMET) [24]. To further screen potential druggability compounds, in silico ADMET model was performed using SwissADME network software to evaluate the top five candidate small molecules with strong binding capacity. As shown in Table 4 and Table S5, under the screening conditions of Lipinski’s rule of five (with a molecular mass less than 500 Da, no more than five hydrogen bond donors, no more than 10 hydrogen bond acceptors, and an octanol–water partition coefficient log P not greater than 5), the compounds Resorcinol monoacetate and Tropisetron were selected as most promising candidate drugs targeting FGF21, which provide a reference for experimental verification in the future.

## 4. Discussion

The FGF21 gene plays an important role in the mechanism of glucose and lipid metabolism and is involved in many metabolic diseases, such as obesity, diabetes, and non-alcoholic fatty liver [4,7,8]. FGF21 is a promising therapeutic target for these metabolic diseases [25,26,27]. This study attempts to reveal characteristics and core active region of camel FGF21 gene promoter, and screen small molecule compounds targeting FGF21 protein, laying the foundation for regulatory research and drug development based on the camel FGF21 gene.

Bioinformatics analysis is an effective means of obtaining sequence features and providing guidance for experimental validation [16,17]. This study analyzed the camel FGF21 promoter and found that the proximal promoter sequence of the camel FGF21 gene has a high similarity to that of humans (Figure 1). The core active regions of camel FGF21 promoter are mainly located in the −445~−612 bp region (Table 1). Coincidentally, this fragment is contained in a long CGI (Figure 2). Actually, CpG dinucleotide sites of ~80% in the bulk genome are methylated, containing 5-methylcytosine (5 mC), a repressive mark associated with long-term gene silencing in vertebrates. CGIs are CpG-rich DNA sequences with hypomethylation compared to the bulk genome, which overlap promoters and are characterized by sequence features that include DNA hypomethylation, elevated CpG and GC content and the presence of transcription factor binding sites [23,28]. The prediction results demonstrated that the segment −400~−800 is an important active region in the camel FGF21 promoter. In addition, camel FGF21 has more and longer CGIs, suggesting a more transcriptional regulatory activity of camel FGF21. Correspondingly, truncated fragments recombinant vectors and luciferase reporter assay determined that camel FGF21 core promoter is located in the 800 bp region upstream of the TSS. An enhancer may exist between −1000 and −2000 bp (Figure 4). More experiments are needed to determine the enhancer position.

Analysis of the transcription factor binding sites indicates that camel FGF21 promoter shared the most transcription factor binding sites with humans and mice (Figure 3) although with significant differences in promoter sequences (Figure 1) and binding site positions in the promoter. The major functional transcription factors such as SP1 [29,30], NF-1 [31], GATA1 [32], YY1 [33] and Elk-1 [34] nearly appeared in all mammals, suggesting that the signaling pathways involved in FGF21 gene transcription regulation are conservative in mammals. However, more work is still needed to identify differential transcription factors that can help reveal the specific regulatory patterns of camel FGF21.

Molecular docking was conducted to screen targeted drugs for proteins and has become an important common component of the drug discovery toolbox [18], which helps to shorten the cycle of new drug development, reduce the cost of drug development, and speed up the drug development process. This study found that the FGF21 protein structure is conserved in mammals and owns a drug-binding pocket (Figure 5 and Table S4). Molecular docking screening found many small compounds showed specific binding ability with FGF21 protein, especially the five molecules, Sorafenib, Resorcinol monoacetate, Sertraline, Tropisetron and Gramicidin D. Previous reports showed that Sorafenib is a known kinase inhibitor with anti-tumor activity [35]. Sertraline is an antidepressant drug and induces multiple cytological effects [36]. Tropisetron, as a serotonin antagonist, affects blood glucose lowering, insulin synthesis, and pancreatic inflammation [37]. Its regulatory function overlaps with FGF21 protein deletion, suggesting that Tropisetron may exert biological effects by affecting the FGF21 protein. Gramicidin D, as an antibacterial peptide, was discovered to have binding activity with many other proteins [38].

Efficacy and safety are considered some of the major causes of clinical attrition during the development of new chemical entities. ADMET (absorption, distribution, metabolism, excretion, and toxicity) describes a drug molecule’s pharmacokinetics and pharmacodynamics properties. ADMET profile of a bioactive compound can impact its efficacy and safety [39]. In silico ADMET platform helped to generate models for a variety of pharmacokinetic and physicochemical endpoints in assisting with the selection and design of novel drugs, as well as the process of drug optimization in the past two decades [40].

Combining molecular docking and ADMET druggability prediction, our study identified Resorcinol monoacetate and Tropisetron as the most promising candidate drugs targeting FGF21. Although more experimental data are still needed to verify their effectiveness, this study provides guidance for studying the specific regulation of the camel FGF21 gene and for drug research targeting the FGF21 protein. Additionally, the recent successful establishment of a Bactrian camel fibroblast cell line, BCF23 [41], also lays the foundation for subsequent experimental research and the expansion of camel-specific metabolic regulation research.

## Figures and Tables

**Table 1:** Core active region of FGF21 gene promoter.

Species	Location/bp	Sequence	Score
Camel	−445~−495	TGCCGCCTGGAACAAAAGCGGCGCTCCTCTGCAGACCTGAAGCGCTTTCC	0.90
	−562~−612	CTGATTGCTGCTTTCTAGAAGGGCACGTCCCGGAGGCGGCTTCTCCGGAG	0.81
Mouse	−18~−68	CCTGTCTGGGTATAAATTCTGGTATTTCTGCGTTCACCAGACAGCCTTAG	0.96
	−2049~−2099	TTCCAAGGTGTACAGCCTCCGCCCTCCCGGCAGGGCAGGCAGCAGGAGGC	0.82

**Table 2:** Major functional transcription factor binding in FGF21 promoter of different mammals.

Transcription Factor	Camel	Mouse	Human	Transcription Factor	Camel	Mouse	Human
SP1	+	+	+	GATA-1	−	−	+
NF-1	+	+	+	USF2	+	+	+
TCF-1	+	−	−	YY1	+	+	+
c-Ets-1	+	−	−	FOXN2	+	−	−
Elk-1	+	+	+	E2F	+	−	+
Pax-4	+	+	+	MyoD	+	+	+

+ and − indicated presence or absence, respectively.

**Table 3:** Physical and chemical property parameters of five drug molecules (The data were obtained from Drugbank).

Drug Name	Relative Molecular Mass	H-Bond Donors	H-Bond Acceptors	Rotatable Bonds	Polar Surface Area (Å2)
Sorafenib	464.825	3	3	6	92.35
Resorcinol monoacetate	152.149	1	2	2	46.53
Sertraline	306.23	1	1	2	12.03
Tropisetron	284.3529	1	2	3	45.33
Gramicidin D	1811.253	20	16	50	519.89

Note: H-bond: hydrogen bond.

**Table 4:** In silico ADMET screening of druggability compounds under Lipinski’s rule of five.

Name	Rotatable Bonds	H-Bond Acceptors	H-Bond Donors	Consensus Log P	GI Absorption	log Kp (cm/s)
Sorafenib	9	7	3	4.1	Low	−6.25
Resorcinol monoacetate	2	3	1	1.32	High	−6.35
Sertraline	2	1	1	4.55	High	−4.77
Tropisetron	3	3	1	2.79	High	−5.53

Note: The druggability analysis of Gramicidin D is failed by SwissADME due to its large molecular mass. H-bond: hydrogen bond. GI absorption: gastrointestinal absorption.

## Data Availability

Not applicable.

## Supplementary Materials

The following supporting information can be downloaded at: https://www.mdpi.com/article/10.3390/vetsci10070452/s1, Table S1: Prediction of transcription factor binding sites in camel FGF21 promoter; Table S2: Prediction of transcription factor binding sites in mouse FGF21 promoter; Table S3: Prediction of transcription factor binding sites in human FGF21 promoter; Table S4: Amino acid residues that form drug binding pockets in FGF21 protein; Table S5: The detailed parameters of druggability compounds under Lipinski’s rule of five using in silico ADMET screening.

## References

[1] Kharitonenkov A., DiMarchi R. (2017). Fibroblast growth factor 21 night watch: Advances and uncertainties in the field. J. Intern. Med..

[2] Agrawal A., Parlee S., Perez-Tilve D., Li P., Pan J., Mroz P.A., Kruse Hansen A.M., Andersen B., Finan B., Kharitonenkov A. (2018). Molecular elements in FGF19 and FGF21 defining KLB/FGFR activity and specificity. Mol. Metab..

[3] Zhou B., Claflin K.E., Flippo K.H., Sullivan A.I., Asghari A., Tadinada S.M., Jensen-Cody S.O., Abel T., Potthoff M.J. (2022). Central FGF21 production regulates memory but not peripheral metabolism. Cell Rep..

[4] Staiger H., Keuper M., Berti L., Hrabe de Angelis M., Haring H.U. (2017). Fibroblast Growth Factor 21-Metabolic Role in Mice and Men. Endocr. Rev..

[5] Minard A.Y., Tan S.X., Yang P., Fazakerley D.J., Domanova W., Parker B.L., Humphrey S.J., Jothi R., Stockli J., James D.E. (2016). mTORC1 Is a Major Regulatory Node in the FGF21 Signaling Network in Adipocytes. Cell Rep..

[6] Engel J.A., Jerlhag E., Svensson L., Smith R.G., Egecioglu E. (2015). Blockade of growth hormone secretagogue receptor 1A signaling by JMV 2959 attenuates the NMDAR antagonist, phencyclidine-induced impairments in prepulse inhibition. Psychopharmacology.

[7] Rajaram R.B., Jayaraman T., Yoong B.K., Koh P.S., Loh P.S., Koong J.K., Khalil A.A., Hashim N., Jamaluddin F.H., Mahadeva S. (2022). Non-alcoholic fatty liver disease and obesity among adult donors are major challenges to living-donor liver transplantation: A single-center experience. Asian J. Surg..

[8] Zheng S., Wu J., Xiang S., Zang Y., Kong D., Wei X., Sun W., Li W. (2022). An fgf21-like gene from swamp eel (Monopterus albus): Recombinant expression and its potential roles in glucose and lipid homeostasis. Comp. Biochem. Physiol. Part. A Mol. Integr. Physiol..

[9] Yanucil C., Kentrup D., Li X., Grabner A., Schramm K., Martinez E.C., Li J., Campos I., Czaya B., Heitman K. (2022). FGF21-FGFR4 signaling in cardiac myocytes promotes concentric cardiac hypertrophy in mouse models of diabetes. Sci. Rep..

[10] Rühlmann C., Dannehl D., Brodtrück M., Adams A.C., Kuhla A. (2021). Neuroprotective Effects of the FGF21 Analogue LY2405319. J. Alzheimer’s Dis. JAD.

[11] Weng Y., Ishino T., Sievers A., Talukdar S., Chabot J.R., Tam A., Duan W., Kerns K., Sousa E., He T. (2018). Glyco-engineered Long Acting FGF21 Variant with Optimal Pharmaceutical and Pharmacokinetic Properties to Enable Weekly to Twice Monthly Subcutaneous Dosing. Sci. Rep..

[12] Hoter A., Rizk S., Naim H.Y. (2019). Cellular and Molecular Adaptation of Arabian Camel to Heat Stress. Front. Genet..

[13] Haberle V., Stark A. (2018). Eukaryotic core promoters and the functional basis of transcription initiation. Nat. Rev. Mol. Cell Biol..

[14] Dao L.T.M., Galindo-Albarran A.O., Castro-Mondragon J.A., Andrieu-Soler C., Medina-Rivera A., Souaid C., Charbonnier G., Griffon A., Vanhille L., Stephen T. (2017). Genome-wide characterization of mammalian promoters with distal enhancer functions. Nat. Genet..

[15] Andersson R., Gebhard C., Miguel-Escalada I., Hoof I., Bornholdt J., Boyd M., Chen Y., Zhao X., Schmidl C., Suzuki T. (2014). An atlas of active enhancers across human cell types and tissues. Nature.

[16] Andersson R., Sandelin A. (2020). Determinants of enhancer and promoter activities of regulatory elements. Nat. Rev. Genet..

[17] Sethi A., Gu M., Gumusgoz E., Chan L., Yan K.K., Rozowsky J., Barozzi I., Afzal V., Akiyama J.A., Plajzer-Frick I. (2020). Supervised enhancer prediction with epigenetic pattern recognition and targeted validation. Nat. Methods.

[18] Stanzione F., Giangreco I., Cole J.C. (2021). Use of molecular docking computational tools in drug discovery. Prog. Med. Chem..

[19] Pinzi L., Rastelli G. (2019). Molecular Docking: Shifting Paradigms in Drug Discovery. Int. J. Mol. Sci..

[20] Gao Y., Zhao S., Zhang W., Tang H., Yan M., Yong F., Bai X., Wu X., Zhang Y., Zhang Q. (2023). Localization of FGF21 Protein and Lipid Metabolism-Related Genes in Camels. Life.

[21] Pollastri M.P. (2010). Overview on the Rule of Five. Curr. Protoc. Pharmacol..

[22] Fisher F.M., Maratos-Flier E. (2016). Understanding the Physiology of FGF21. Annu. Rev. Physiol..

[23] Hughes A.L., Szczurek A.T., Kelley J.R., Lastuvkova A., Turberfield A.H., Dimitrova E., Blackledge N.P., Klose R.J. (2023). A CpG island-encoded mechanism protects genes from premature transcription termination. Nat. Commun..

[24] Ferreira L.L.G., Andricopulo A.D. (2019). ADMET modeling approaches in drug discovery. Drug Discov. Today.

[25] BonDurant L.D., Potthoff M.J. (2018). Fibroblast Growth Factor 21: A Versatile Regulator of Metabolic Homeostasis. Annu. Rev. Nutr..

[26] Tezze C., Romanello V., Sandri M. (2019). FGF21 as Modulator of Metabolism in Health and Disease. Front. Physiol..

[27] Kim J.H., Lim S., Seo M., Choi H.H., Kim D., Mi K.J., Park J.Y., Choi B.H., Lee J.K., Kim J.G. (2022). Long-Acting FGF21 Fusion Proteins and Pharmaceutical Composition Comprising Same. U.S. Patent.

[28] Angeloni A., Bogdanovic O. (2021). Sequence determinants, function, and evolution of CpG islands. Biochem. Soc. Trans..

[29] Yu Q., Liu W., Chen Z., Zhang M. (2021). Specificity Protein 1: A Protein With a Two-Sided Role in Ischemic Stroke. Front. Cell. Neurosci..

[30] Laniel M.A., Poirier G.G., Guerin S.L. (2001). Nuclear factor 1 interferes with Sp1 binding through a composite element on the rat poly(ADP-ribose) polymerase promoter to modulate its activity in vitro. J. Biol. Chem..

[31] Gutmann D.H., Ferner R.E., Listernick R.H., Korf B.R., Wolters P.L., Johnson K.J. (2017). Neurofibromatosis type 1. Nat. Rev. Dis. Primers.

[32] Steiner L. (2022). Helping GATA1 make complex decisions. Blood.

[33] Martins Pecanha F.L., Jaafar R., Werneck-de-Castro J.P., Apostolopolou C.C., Bhushan A., Bernal-Mizrachi E. (2022). The Transcription Factor YY1 Is Essential for Normal DNA Repair and Cell Cycle in Human and Mouse beta-Cells. Diabetes.

[34] Quintero-Barceinas R.S., Gehringer F., Ducker C., Saxton J., Shaw P.E. (2021). ELK-1 ubiquitination status and transcriptional activity are modulated independently of F-Box protein FBXO25. J. Biol. Chem..

[35] Kong F.H., Ye Q.F., Miao X.Y., Liu X., Huang S.Q., Xiong L., Wen Y., Zhang Z.J. (2021). Current status of sorafenib nanoparticle delivery systems in the treatment of hepatocellular carcinoma. Theranostics.

[36] Hwang H.Y., Shim J.S., Kim D., Kwon H.J. (2021). Antidepressant drug sertraline modulates AMPK-MTOR signaling-mediated autophagy via targeting mitochondrial VDAC1 protein. Autophagy.

[37] Naderi R., Shirpoor A., Samadi M., Pourheydar B., Moslehi A. (2020). Tropisetron attenuates pancreas apoptosis in the STZ-induced diabetic rats: Involvement of SIRT1/NF-kappaB signaling. Pharmacol. Rep..

[38] Protic S., Kalicanin N., Sencanski M., Prodanovic O., Milicevic J., Perovic V., Paessler S., Prodanovic R., Glisic S. (2023). In Silico and In Vitro Inhibition of SARS-CoV-2 PL(pro) with Gramicidin D. Int. J. Mol. Sci..

[39] Jia L., Gao H. (2022). Machine Learning for In Silico ADMET Prediction. Methods Mol. Biol..

[40] Goller A.H., Kuhnke L., Montanari F., Bonin A., Schneckener S., Ter Laak A., Wichard J., Lobell M., Hillisch A. (2020). Bayer’s in silico ADMET platform: A journey of machine learning over the past two decades. Drug Discov. Today.

[41] Yan M., Yong F., Ji W., Zhang L., Zhao S., Gao Y. (2023). Construction and Characterization of Immortalized Fibroblast Cell Line from Bactrian Camel. Life.

