# Supplementary material for: Analysis of Functional Promoter of Camel FGF21 Gene and Identification of Small Compounds Targeting FGF21 Protein
Source: Vet Sci. 2023 Jul 10;10(7):452. doi: 10.3390/vetsci10070452 (PMC10383868; doi:10.3390/vetsci10070452)
Supplement: Supplementary file 1 [file vetsci-10-00452-s001.zip › suppl materials.pdf]

Supplementary data

Table S1 Prediction of transcription factor binding sites in camel FGF21 promoter

Transcription factor	Start site/bp	Strand	Binding sequence
HNF-4	-1671	+	TGAGGGGGAAAGTCCATCA
Elk-1	-1647	+	GTGACAGGAAGTGACA
Elk-1	-1549	-	AGACTTCCGGGGTC
c-Ets-1(p54)	-1548	-	GACTTCCGGG
Oct-1	-781	+	GCGGATGCAAAACCT
E2F	-730	-	GCGCGAAA
Pax-4	-624	-	GGGGTCTTACGCCTGATTGCT
Pax-6	-623	+	GGGTCTTACGCCTGATTGCTG
v-Myb	-339	-	GACCGTTATT
NF-1	-248	+	CACCCTTAGATTCTGGGAAATTGCCACTC

Table S2 Prediction of transcription factor binding sites in mouse FGF21 promoter

Transcription factor	Start site/bp	Strand	Binding sequence
NF-1	-2110	-	GAGGGGCAGGGTTCCAAGGTGTACAGCCT
CAR1	-1658	+	TTGTAGCCGCCTACACA
Pax-6	-879	-	GCACCTTGAAGCTTAAAATTC
Hand1/E47	-785	-	AGAGCCAGACCCTAGC
Hand1/E47	-719	-	AGAGCCAGACCCTAGC
C-Re1	-184	-	GGAAAGACCC
Pax-4	-101	+	ACGCGTCAGGAGTGGGGAGGG
USF	-81	-	GCACGTGG

Table S3 Prediction of transcription factor binding sites in human FGF21 promoter

Transcription factor	Start site/bp	Strand	Binding sequence
USF	-1782	+	GTCACGTGAT
USF	-1782	-	GTCACGTGAT
SREBP-1	-1782	-	GTCACGTGATC
CHOP-C/EBPalpha	-1436	-	GGAGATTGCAGTG
HSF	-1159	+	AGAAAAGGAGATTCC
v-Myb	-1128	+	AGTAACGGGT
Pax-4	-1104	-	TGGCGTTCCCGCTTGACCAAG
HNF-1	-934	+	AGCTAATGATTAATAAT
Evi-1	-862	-	TAACTATTCTTATTC
BR-C Z4	-841	-	TTTTCTTTATTAT
CRP	-726	-	GAGTTTGACTTGATTCCCAGAAGTTT
HNF-4	-453	+	TCCAGACCAAAGCTCCCTC

Table S4 Amino acid residues that form drug binding pockets in FGF21 protein

Camel	Human	Mouse
Leu-165	Arg-163	Ala-60
Ile-91	His-60	Thr-58
Gly-41	Lys-150	Ala-74
Gln-43	Ala-72	Lys-150
Phe-123	Leu-144	Gly-72
Pro-166	Ala-73	Ser-152
Pro-88	Gly-70	His-61
Tyr-132	Ala-162	Ala-73
Ala-85	Glu-58	Leu-62
Gly-131	Gly-148	Val-71
Leu-38	Pro-152	Val-70
Gln-124	Asp-53	Asp-55
Gly-42	Ser-151	Asp-53
Leu-127	Leu-146	Asp-54
Gln-39	Arg-64	Trp-160
Glu-125	His-145	Val-163
Leu-126	Arg-159	Arg-146
Lys-87	Thr-51	Gln-149
Leu-86	Ala-54	Glu-59
Pro-36	Gln-55	Gly-161
Arg-45	Val-69	Gln-56
Arg-47	Gln-56	Asp-151
Leu-128	Asn-149	Thr-52
Leu-167	Gly-160	Asp-57
Gly-89	Asp-52	Leu-147
Asn-133	Pro-161	Pro-153
Phe-164	Phe-164	Pro-148
Leu-83	Ala-59	Arg-65
Lys-84	Gly-71	
Pro-168	Thr-57	
Phe-40	Leu-61	
Gln-92	Pro-147	
Val-90		
Tyr-135		
Val-44		
Leu-37		

Table S5 The first 1000 small molecules with binding ability to camel FGF21 protein

Dataset_ID	Generic_Name	Dock_Score
DB00398	Sorafenib	-80.214539
DB15480	Resorcinol monoacetate	-80.094124
DB01104	Sertraline	-79.782677
DB11699	Tropisetron	-79.740334
DB00027	Gramicidin D	-78.900314
DB00080	Daptomycin	-78.730309
DB06152	Nylidrin	-77.349968
DB09216	Tolfenamic acid	-76.053978
DB00007	Leuprolide	-75.349083
DB09118	Stiripentol	-75.342964
DB00639	Butoconazole	-74.700356
DB13970	Dioctyldimonium	-74.527985
DB11274	Dihydro-alpha-ergocryptine	-74.464798
DB12401	Bromperidol	-71.047447
DB15328	Ubrogepant	-70.18634
DB00564	Carbamazepine	-68.927849
DB11269	Diethylamino hydroxybenzoyl hexyl benzoate	-68.528175
DB13981	Nomegestrol acetate	-68.397385
DB14219	Monomethyl fumarate	-65.756195
DB14761	Remdesivir	-65.460068
DB06794	Lodoxamide	-65.346268
DB11757	Istradefylline	-65.011703
DB08872	Gabapentin enacarbil	-64.1577
DB00560	Tigecycline	-63.923641
DB13074	Macimorelin	-63.622601
DB11751	Cabotegravir	-63.092083
DB09351	Levobetaxolol	-62.531593
DB11235	Thonzylamine	-61.16555
DB13909	Bismuth subgallate	-60.608559
DB02300	Calcipotriol	-60.587734
DB06788	Histrelin	-60.563145
DB00180	Flunisolide	-59.825279
DB06795	Mafenide	-59.384697
DB01265	Telbivudine	-59.159863
DB09257	Gimeracil	-59.057758
DB09026	Aliskiren	-59.043941
DB00775	Tirofiban	-58.940495
DB00861	Diflunisal	-58.663059
DB06694	Xylometazoline	-58.349384
DB03166	Acetic acid	-57.788406
DB00724	Imiquimod	-57.373276
DB09048	Netupitant	-57.137642

DB00513	Aminocaproic acid	-57.090767
DB00621	Oxandrolone	-57.066841
DB09342	Propoxycaine	-56.993771
DB08973	Fluclorolone acetonide	-56.541836
DB11326	Boric acid	-56.497417
DB00797	Tolazoline	-56.122505
DB09085	Tetracaine	-55.949715
DB09385	Cyanocobalamin Co-57	-55.716141
DB08871	Eribulin	-55.603371
DB02925	Piretanide	-55.369366
DB08930	Dolutegravir	-55.259151
DB11331	1-Palmitoyl-2-oleoyl-sn-glycero-3-(phospho-rac-(1-glycerol))	-55.051907
DB06636	Isavuconazonium	-54.979881
DB00014	Goserelin	-54.745239
DB14002	D-alpha-Tocopherol acetate	-54.473118
DB06780	Desoxycorticosterone acetate	-54.357822
DB04876	Vildagliptin	-54.156628
DB06335	Saxagliptin	-53.756943
DB14528	Chromium gluconate	-53.646736
DB09039	Eliglustat	-53.41861
DB00498	Phenindione	-53.3615
DB01065	Melatonin	-53.24342
DB00740	Riluzole	-53.159206
DB14644	Methylprednisolone hemisuccinate	-52.904873
DB06605	Apixaban	-52.778557
DB11182	Rose bengal	-52.369289
DB01243	Chloroxine	-52.358883
DB01219	Dantrolene	-52.356125
DB11123	Hypochlorite	-52.194202
DB09088	Amylocaine	-51.500584
DB14084	Butylparaben	-51.496452
DB14879	Cefiderocol	-51.464638
DB04842	Fluspirilene	-51.371166
DB06594	Agomelatine	-51.310806
DB11157	Anthralin	-51.271233
DB00050	Cetrorelix	-50.895607
DB01135	Doxacurium	-50.600811
DB09477	Enalaprilat	-50.501976
DB00533	Rofecoxib	-50.343235
DB01226	Mivacurium	-50.273956
DB09027	Ledipasvir	-50.273037
DB11145	Oxyquinoline	-50.124191
DB11610	Oxilofrine	-50.123196

DB01158	Bretylium	-50.063786
DB06774	Capsaicin	-50.022469
DB14554	Dotatate	-49.988705
DB05246	Methsuximide	-49.835789
DB00504	Levallorphan	-49.710197
DB08890	Linaclotide	-49.655586
DB11633	Isavuconazole	-49.553635
DB14631	Prednisolone phosphate	-49.449093
DB00309	Vindesine	-49.388508
DB00152	Thiamine	-49.387722
DB01255	Lisdexamfetamine	-49.358395
DB01413	Cefepime	-49.324043
DB06410	Doxercalciferol	-49.202972
DB11936	Bempedoic acid	-49.035202
DB00523	Alitretinoin	-48.845112
DB00938	Salmeterol	-48.826954
DB00790	Perindopril	-48.806641
DB00358	Mefloquine	-48.644421
DB08826	Deferiprone	-48.55228
DB00384	Triamterene	-48.33419
DB00478	Rimantadine	-48.138725
DB01070	Dihydrotachysterol	-48.135902
DB08971	Fluocortolone	-48.126698
DB11967	Binimetinib	-48.02631
DB01320	Fosphenytoin	-48.021084
DB08804	Nandrolone decanoate	-47.818283
DB01359	Penbutolol	-47.729149
DB00281	Lidocaine	-47.685371
DB09133	Iothalamic acid	-47.594761
DB01242	Clomipramine	-47.58886
DB01591	Solifenacin	-47.452888
DB11197	Ferrous cysteine glycinate	-47.332611
DB06228	Rivaroxaban	-47.297688
DB01204	Mitoxantrone	-47.281235
DB11613	Velpatasvir	-47.177097
DB01380	Cortisone acetate	-47.157803
DB00737	Meclizine	-47.139465
DB11712	Tezacaftor	-47.104527
DB00221	Isoetharine	-46.996105
DB12343	Temocillin	-46.714645
DB01146	Diphenylpyraline	-46.705147
DB15477	Alloin	-46.662113
DB04571	Trioxsalen	-46.520042
DB05812	Abiraterone	-46.495499

DB11761	Tenapanor	-46.452267
DB12877	Oxatomide	-46.325535
DB00465	Ketorolac	-46.260048
DB01322	Kava	-46.230057
DB09270	Ubidecarenone	-46.216312
DB00404	Alprazolam	-46.213902
DB06249	Arzoxifene	-46.207577
DB08604	Triclosan	-46.159286
DB00446	Chloramphenicol	-46.139633
DB11359	Guaiacol	-46.133747
DB00963	Bromfenac	-46.067879
DB06262	Droxidopa	-46.065407
DB11951	Lemborexant	-46.046284
DB08981	Fenbufen	-45.903683
DB00217	Bethanidine	-45.815739
DB11619	Gestrinone	-45.809525
DB00941	Hexafluronium	-45.760498
DB11641	Vinflunine	-45.627987
DB11837	Osilodrostat	-45.580875
DB12010	Fostamatinib	-45.547947
DB09267	Strontium ranelate	-45.529865
DB11125	Benzethonium	-45.513889
DB09069	Trimetazidine	-45.508247
DB11630	Temoporfin	-45.444824
DB01116	Trimethaphan	-45.370872
DB09042	Tedizolid phosphate	-45.332275
DB01250	Olsalazine	-45.285816
DB00455	Loratadine	-45.26762
DB00503	Ritonavir	-45.23571
DB01296	Glucosamine	-45.211712
DB01222	Budesonide	-45.199562
DB09195	Lorpiprazole	-45.159317
DB00487	Pefloxacin	-45.139557
DB11859	Brexanolone	-45.12051
DB05273	Samarium (153Sm) lexidronam	-45.058575
DB00296	Ropivacaine	-45.025467
DB14895	Vibegron	-45.005272
DB13848	Fluorodopa (18F)	-44.986897
DB08958	Hexetidine	-44.974827
DB09543	Methyl salicylate	-44.886459
DB00969	Alosetron	-44.85635
DB00380	Dexrazoxane	-44.811966
DB09425	Indium In-111 pentetate	-44.802525
DB12808	Trifarotene	-44.789009

DB00924	Cyclobenzaprine	-44.788349
DB11915	Valbenazine	-44.612083
DB00888	Mechlorethamine	-44.568146
DB12407	Iobitridol	-44.565815
DB06707	Levonordefrin	-44.564377
DB00425	Zolpidem	-44.553905
DB11979	Elagolix	-44.492374
DB06753	Triclofos	-44.489532
DB11660	Latanoprostene bunod	-44.475304
DB13337	Pheneticillin	-44.440418
DB00712	Flurbiprofen	-44.436623
DB11327	Dipyrithione	-44.42902
DB09219	Bisoxatin	-44.421875
DB04868	Nilotinib	-44.374367
DB13931	Netarsudil	-44.287483
DB11570	Padimate O	-44.28476
DB00264	Metoprolol	-44.194847
DB00944	Demecarium	-44.171043
DB11760	Talazoparib	-44.102924
DB00432	Trifluridine	-44.066746
DB00917	Dinoprostone	-44.045574
DB00675	Tamoxifen	-44.017944
DB13114	Amitriptylinoxide	-44.000996
DB00553	Methoxsalen	-43.995613
DB11842	Angiotensin II	-43.882366
DB00931	Metacycline	-43.855515
DB07776	Flavone	-43.823772
DB04845	Ixabepilone	-43.813026
DB00695	Furosemide	-43.783287
DB00226	Guanadrel	-43.76469
DB13345	Dihydroergocristine	-43.758648
DB13213	Butaperazine	-43.742813
DB00872	Conivaptan	-43.733929
DB12602	Pentetreotide	-43.717159
DB11278	DL-Methylephedrine	-43.69413
DB00439	Cerivastatin	-43.615044
DB14840	Ripretinib	-43.533672
DB11750	Clobetasol	-43.48774
DB14669	Betamethasone phosphate	-43.281467
DB09268	Picosulfuric acid	-43.281269
DB04846	Celiprolol	-43.277744
DB00474	Methohexital	-43.25005
DB13265	Hexobendine	-43.173332
DB08797	Salicylamide	-43.156452

DB08877	Ruxolitinib	-43.153656
DB00222	Glimepiride	-43.133072
DB00612	Bisoprolol	-43.127708
DB01256	Retapamulin	-43.101498
DB00283	Clemastine	-43.068737
DB01626	Pargyline	-42.999702
DB00565	Cisatracurium	-42.983265
DB01195	Flecainide	-42.977177
DB01165	Ofloxacin	-42.904625
DB11560	Lesinurad	-42.855183
DB01127	Econazole	-42.843739
DB00942	Cycrimine	-42.823997
DB01409	Tiotropium	-42.822426
DB11989	Benznidazole	-42.801838
DB00543	Amoxapine	-42.767815
DB00597	Gadoteridol	-42.709904
DB00656	Trazodone	-42.678028
DB00200	Hydroxocobalamin	-42.656124
DB01077	Etidronic acid	-42.651924
DB00337	Pimecrolimus	-42.626854
DB13944	Testosterone enanthate	-42.568668
DB11299	Vanillyl butyl ether	-42.54137
DB13858	Dimazole	-42.519993
DB12147	Erdafitinib	-42.481049
DB13943	Testosterone cypionate	-42.377171
DB15305	Risdiplam	-42.359459
DB08824	Ioflupane I-123	-42.323158
DB01205	Flumazenil	-42.321774
DB00153	Ergocalciferol	-42.257423
DB00648	Mitotane	-42.205292
DB06814	Protokylol	-42.123375
DB00855	Aminolevulinic acid	-42.116222
DB00968	Methyldopa	-42.089485
DB14703	Dexamethasone metasulfobenzoate	-42.082592
DB00035	Desmopressin	-42.081631
DB12839	Pegvaliase	-42.054295
DB01599	Probucol	-42.010357
DB09076	Umeclidinium	-41.97266
DB06589	Pazopanib	-41.915936
DB00664	Sulfametopyrazine	-41.887474
DB09280	Lumacaftor	-41.867973
DB07565	Chloramphenicol succinate	-41.840382
DB04115	Berberine	-41.836189
DB00289	Atomoxetine	-41.823711

DB00685	Trovafloxacin	-41.794807
DB01090	Pentolinium	-41.752037
DB08988	Ethoheptazine	-41.746555
DB11656	Rebamipide	-41.727638
DB00253	Medrysone	-41.727024
DB01598	Imipenem	-41.704132
DB05109	Trabectedin	-41.688251
DB08867	Ulipristal	-41.661716
DB01044	Gatifloxacin	-41.648838
DB00934	Maprotiline	-41.640194
DB08897	Aclidinium	-41.5952
DB08905	Formestane	-41.575016
DB15035	Zanubrutinib	-41.567375
DB00772	Malathion	-41.557587
DB14583	Segesterone acetate	-41.538311
DB06813	Pralatrexate	-41.450428
DB00617	Paramethadione	-41.44252
DB00959	Methylprednisolone	-41.428448
DB06796	Mangafodipir	-41.421535
DB00274	Cefmetazole	-41.41301
DB00201	Caffeine	-41.399746
DB13947	Testosterone enantate benzilic acid hydrazone	-41.364323
DB08895	Tofacitinib	-41.323807
DB00680	Moricizine	-41.31086
DB01582	Sulfamethazine	-41.286568
DB04339	Carbocisteine	-41.261127
DB13286	Bumadizone	-41.236034
DB11986	Entrectinib	-41.174496
DB06212	Tolvaptan	-41.165249
DB00351	Megestrol acetate	-41.15361
DB00755	Tretinoin	-41.142647
DB01020	Isosorbide mononitrate	-41.103687
DB01112	Cefuroxime	-41.084396
DB00315	Zolmitriptan	-41.019451
DB01153	Sertaconazole	-41.016029
DB14184	Cinnamaldehyde	-41.000042
DB00558	Zanamivir	-40.985401
DB01142	Doxepin	-40.981335
DB08907	Canagliflozin	-40.964943
DB11386	Chlorobutanol	-40.945557
DB04572	Thiotepa	-40.933495
DB00134	Methionine	-40.889076
DB13711	Tritoqualine	-40.850628
DB13707	Sodium tartrate	-40.742477

DB14200	Thiohexam	-40.717655
DB00248	Cabergoline	-40.71632
DB06608	Tafenoquine	-40.702038
DB08329	Sulthiame	-40.66497
DB00219	Oxyphenonium	-40.643299
DB01016	Glyburide	-40.632008
DB00627	Niacin	-40.612347
DB00769	Hydrocortamate	-40.604996
DB00537	Ciprofloxacin	-40.583694
DB03312	Brivudine	-40.567814
DB01068	Clonazepam	-40.523117
DB06176	Romidepsin	-40.505283
DB00716	Nedocromil	-40.484013
DB01638	Sorbitol	-40.475063
DB08882	Linagliptin	-40.440147
DB09064	Ciprofibrate	-40.438286
DB00645	Dyclonine	-40.426834
DB04880	Enoximone	-40.425301
DB03128	Acetylcholine	-40.412094
DB11115	Ensulizole	-40.388206
DB09272	Eluxadoline	-40.34502
DB00304	Desogestrel	-40.250896
DB00632	Docosanol	-40.225239
DB13878	Pibrentasvir	-40.207348
DB11800	Tivozanib	-40.197388
DB11336	Kinetin	-40.188412
DB00766	Clavulanic acid	-40.159142
DB01346	Quinidine barbiturate	-40.140194
DB01406	Danazol	-40.136898
DB01319	Fosamprenavir	-40.108711
DB01247	Isocarboxazid	-40.089703
DB00871	Terbutaline	-40.073105
DB09242	Moxonidine	-40.063583
DB00609	Ethionamide	-40.027645
DB01744	Camphor	-40.024067
DB11590	Thimerosal	-40.022549
DB11901	Apalutamide	-40.021194
DB06698	Betahistine	-40.015423
DB00997	Doxorubicin	-40.010284
DB11431	Moxidectin	-39.988983
DB01074	Perhexiline	-39.988266
DB01010	Edrophonium	-39.972786
DB11190	Pantethine	-39.956524
DB08987	Etidocaine	-39.947662

DB14598	Edetate calcium disodium anhydrous	-39.941418
DB00170	Menadione	-39.934509
DB12245	Triclabendazole	-39.931293
DB01061	Azlocillin	-39.846542
DB00525	Tolnaftate	-39.842083
DB14475	L-Lactic acid	-39.840927
DB00792	Tripelennamine	-39.84016
DB00699	Nicergoline	-39.80867
DB09343	Tipiracil	-39.725323
DB12278	Propiverine	-39.712959
DB09070	Tibolone	-39.691132
DB01231	Diphenidol	-39.677341
DB09015	Canrenoic acid	-39.651306
DB09115	Diiodohydroxyquinoline	-39.632332
DB01400	Neostigmine	-39.628471
DB00203	Sildenafil	-39.623138
DB00870	Suprofen	-39.594563
DB09114	Colfosceril palmitate	-39.515232
DB11609	Normethadone	-39.493069
DB12364	Betrixaban	-39.486008
DB00898	Ethanol	-39.47805
DB00313	Valproic acid	-39.474155
DB13838	Noxytiolin	-39.401775
DB01092	Ouabain	-39.391418
DB01198	Zopiclone	-39.378181
DB11820	Nifurtimox	-39.364639
DB01171	Moclobemide	-39.363853
DB06811	Polidocanol	-39.347244
DB00781	Polymyxin B	-39.332092
DB11071	Phenyl salicylate	-39.319244
DB09213	Dexibuprofen	-39.314804
DB08873	Boceprevir	-39.297699
DB08933	Luliconazole	-39.293068
DB11219	Enzacamene	-39.242451
DB03619	Deoxycholic acid	-39.230572
DB04824	Phenolphthalein	-39.221218
DB09376	Lapyrium	-39.201473
DB12267	Brigatinib	-39.191822
DB01022	Phylloquinone	-39.18116
DB12499	Clascoterone	-39.175343
DB09061	Cannabidiol	-39.173721
DB00599	Thiopental	-39.162094
DB00491	Miglitol	-39.126343
DB01612	Amyl Nitrite	-39.120583

DB01324	Polythiazide	-39.092461
DB00922	Levosimendan	-39.088226
DB09134	Ioversol	-39.081772
DB09056	Amorolfine	-39.076424
DB06274	Alvimopan	-39.075413
DB12070	Letermovir	-39.046734
DB01628	Etoricoxib	-39.022118
DB01420	Testosterone propionate	-38.989838
DB01054	Nitrendipine	-38.983269
DB01604	Pivampicillin	-38.981789
DB11642	Pitolisant	-38.965134
DB01245	Decamethonium	-38.925938
DB05541	Brivaracetam	-38.799248
DB00299	Penciclovir	-38.797012
DB00476	Duloxetine	-38.796726
DB14677	Gestonorone caproate	-38.778667
DB01060	Amoxicillin	-38.777294
DB00440	Trimethoprim	-38.751633
DB00875	Flupentixol	-38.7425
DB09099	Somatostatin	-38.72789
DB00760	Meropenem	-38.719086
DB12532	Oxetacaine	-38.70657
DB14489	Ferrous succinate	-38.70475
DB01333	Cefradine	-38.659237
DB00424	Hyoscyamine	-38.649704
DB00174	Asparagine	-38.592186
DB01403	Methotrimeprazine	-38.566578
DB00388	Phenylephrine	-38.544868
DB00316	Acetaminophen	-38.499283
DB12377	Relebactam	-38.451435
DB14539	Hydrocortisone acetate	-38.443146
DB12293	Nefopam	-38.437565
DB00732	Atracurium besylate	-38.431976
DB00485	Dicloxacillin	-38.422501
DB01076	Atorvastatin	-38.408413
DB13783	Acemetacin	-38.396828
DB08868	Fingolimod	-38.371273
DB08881	Vemurafenib	-38.367954
DB08827	Lomitapide	-38.330078
DB00185	Cevimeline	-38.328171
DB09110	Coenzyme M	-38.327065
DB11637	Delamanid	-38.324017
DB03796	Palmitic Acid	-38.310543
DB01187	Iophendylate	-38.30484

DB13225	Dibenzepin	-38.251526
DB00666	Nafarelin	-38.244743
DB06216	Asenapine	-38.181175
DB11640	Amifampridine	-38.179443
DB00584	Enalapril	-38.174343
DB11594	Domiphen	-38.167885
DB11586	Asunaprevir	-38.162151
DB12554	Mebeverine	-38.160671
DB06413	Armodafinil	-38.157688
DB09502	Fludeoxyglucose (18F)	-38.156258
DB00211	Midodrine	-38.138668
DB03085	Glycolic acid	-38.119427
DB06196	Icatibant	-38.116959
DB00762	Irinotecan	-38.101929
DB06716	Fospropofol	-38.080181
DB00271	Diatrizoate	-38.076122
DB00488	Altretamine	-38.066376
DB09132	Gadoteric acid	-38.066212
DB00682	Warfarin	-38.052208
DB00636	Clofibrate	-38.04174
DB04908	Flibanserin	-38.033291
DB11387	Chloroform	-38.028908
DB05219	Crisaborole	-38.017567
DB00906	Tiagabine	-38.00164
DB00156	Threonine	-37.987225
DB01105	Sibutramine	-37.918182
DB00369	Cidofovir	-37.86417
DB01041	Thalidomide	-37.824051
DB09020	Bisacodyl	-37.815468
DB00342	Terfenadine	-37.777309
DB00536	Guanidine	-37.764156
DB12945	Dihydralazine	-37.763222
DB14202	Morpholinylmercaptobenzothiazole	-37.753105
DB13346	Bufexamac	-37.751877
DB01331	Cefoxitin	-37.71439
DB12674	Lurbinectedin	-37.711033
DB11827	Ertugliflozin	-37.710903
DB12329	Eravacycline	-37.681797
DB00688	Mycophenolate mofetil	-37.657696
DB04835	Maraviroc	-37.625256
DB00796	Candesartan cilexetil	-37.61652
DB08936	Chlorcyclizine	-37.600887
DB00272	Betazole	-37.600033
DB01291	Pirbuterol	-37.585083

DB00690	Flurazepam	-37.579041
DB00557	Hydroxyzine	-37.54924
DB00159	Icosapent	-37.506035
DB01039	Fenofibrate	-37.499557
DB01595	Nitrazepam	-37.479111
DB04840	Debrisoquine	-37.459061
DB01212	Ceftriaxone	-37.458912
DB09237	Levamlodipine	-37.447262
DB04948	Lofexidine	-37.431278
DB11201	Menthyl salicylate	-37.379311
DB09292	Sacubitril	-37.375893
DB06403	Ambrisentan	-37.364079
DB01303	Oxtriphylline	-37.362961
DB13421	Edoxudine	-37.272045
DB00672	Chlorpropamide	-37.213715
DB01623	Thiothixene	-37.209732
DB00339	Pyrazinamide	-37.179356
DB00512	Vancomycin	-37.17379
DB12466	Favipiravir	-37.151222
DB11207	Amiloxate	-37.14983
DB11284	DL-dimyristoylphosphatidylglycerol	-37.142044
DB08820	Ivacaftor	-37.140247
DB03808	Hexamidine	-37.135391
DB06827	Viomycin	-37.132984
DB11815	Tilarginine	-37.131245
DB00912	Repaglinide	-37.113609
DB00333	Methadone	-37.11348
DB06614	Peramivir	-37.100674
DB04864	Huperzine A	-37.073231
DB12500	Fedratinib	-37.070759
DB09059	Atosiban	-37.068722
DB14643	Methylprednisolone aceponate	-37.05962
DB00865	Benzphetamine	-37.055199
DB00857	Terbinafine	-37.020168
DB00175	Pravastatin	-37.015129
DB00236	Pipobroman	-37.010818
DB00196	Fluconazole	-36.992802
DB11828	Neratinib	-36.970722
DB01128	Bicalutamide	-36.947052
DB14512	Mometasone furoate	-36.888649
DB01233	Metoclopramide	-36.877331
DB14649	Dexamethasone acetate	-36.853554
DB01194	Brinzolamide	-36.843544
DB14569	Tedizolid	-36.83746

DB01174	Phenobarbital	-36.829987
DB01613	Erythrityl tetranitrate	-36.81057
DB08815	Lurasidone	-36.809628
DB00216	Eletriptan	-36.783035
DB11181	Homatropine	-36.767345
DB08860	Pitavastatin	-36.76247
DB11217	Arbutin	-36.74868
DB04890	Bepotastine	-36.745071
DB00579	Mazindol	-36.736088
DB00224	Indinavir	-36.735699
DB13624	Methoxyphenamine	-36.712234
DB01157	Trimetrexate	-36.704319
DB13911	Phloxine B	-36.688728
DB08944	Isoaminile	-36.678165
DB00521	Carteolol	-36.649281
DB00240	Alclometasone	-36.641891
DB01037	Selegiline	-36.638351
DB12328	Cantharidin	-36.632442
DB14674	Estramustine phosphate	-36.62315
DB11942	Selinexor	-36.612396
DB01618	Molindone	-36.57095
DB01178	Chlormezanone	-36.56052
DB14596	Loteprednol etabonate	-36.541447
DB00876	Eprosartan	-36.533791
DB11629	Laropiprant	-36.532806
DB09060	Avibactam	-36.52779
DB01589	Quazepam	-36.515911
DB05676	Apremilast	-36.495789
DB13142	Calcium glubionate anhydrous	-36.477936
DB09101	Elvitegravir	-36.472538
DB00305	Mitomycin	-36.472507
DB06154	Pentaerythritol tetranitrate	-36.442055
DB01186	Pergolide	-36.437611
DB06824	Triethylenetetramine	-36.431763
DB00966	Telmisartan	-36.405106
DB01620	Pheniramine	-36.36895
DB09183	Dasabuvir	-36.367229
DB11206	Bemotrizinol	-36.36462
DB09185	Viloxazine	-36.361519
DB13180	Gluconic Acid	-36.357841
DB04816	Dantron	-36.35054
DB01082	Streptomycin	-36.329304
DB01057	Echothiophate	-36.313873
DB03929	D-Serine	-36.307785

DB06230	Nalmefene	-36.301941
DB11614	Rupatadine	-36.298717
DB06713	Norelgestromin	-36.280685
DB01329	Cefoperazone	-36.280514
DB13972	Racemethionine	-36.273621
DB09320	Procaine benzylpenicillin	-36.272884
DB11703	Acalabrutinib	-36.254803
DB01183	Naloxone	-36.252937
DB06802	Nepafenac	-36.250629
DB08823	Spinosad	-36.246067
DB08822	Azilsartan medoxomil	-36.244671
DB00106	Abarelix	-36.232079
DB06237	Avanafil	-36.226692
DB13801	Muzolimine	-36.211758
DB12097	Mannitol busulfan	-36.20356
DB00554	Piroxicam	-36.174625
DB04822	Oxeladin	-36.161682
DB01328	Cefonicid	-36.149292
DB00633	Dexmedetomidine	-36.147457
DB08887	Icosapent ethyl	-36.144432
DB00820	Tadalafil	-36.094585
DB01147	Cloxacillin	-36.090492
DB08875	Cabozantinib	-36.055378
DB08896	Regorafenib	-36.0504
DB13956	Estradiol valerate	-36.022038
DB06201	Rufinamide	-36.01569
DB08932	Macitentan	-35.979824
DB05154	Pretomanid	-35.979572
DB06207	Silodosin	-35.97953
DB09136	Isosulfan blue	-35.977203
DB09149	Florbetapir (18F)	-35.969437
DB00128	Aspartic acid	-35.949871
DB01326	Cefamandole	-35.947514
DB13955	Estradiol dienanthate	-35.927765
DB09119	Eslicarbazepine acetate	-35.908691
DB09146	Iron sucrose	-35.901012
DB08903	Bedaquiline	-35.900272
DB00618	Demeclocycline	-35.893631
DB01238	Aripiprazole	-35.879021
DB14658	Chloramphenicol palmitate	-35.869633
DB00137	Lutein	-35.867516
DB13246	Quinupramine	-35.854954
DB01125	Anisindione	-35.851391
DB00385	Valrubicin	-35.833347

DB11279	Brilliant green cation	-35.831207
DB01087	Primaquine	-35.830723
DB13222	Tilbroquinol	-35.821873
DB01401	Choline magnesium trisalicylate	-35.809017
DB11636	Nomegestrol	-35.806923
DB00698	Nitrofurantoin	-35.795845
DB08918	Levomilnacipran	-35.786781
DB11793	Niraparib	-35.770947
DB06689	Ethanolamine oleate	-35.767086
DB06203	Alogliptin	-35.763058
DB06448	Lonafarnib	-35.758305
DB01001	Salbutamol	-35.75016
DB11226	Ethylhexyl methoxycrylene	-35.74931
DB00129	Ornithine	-35.728493
DB12141	Gilteritinib	-35.728371
DB06736	Aceclofenac	-35.720264
DB06401	Bazedoxifene	-35.715954
DB00276	Amsacrine	-35.699265
DB00948	Mezlocillin	-35.687992
DB00393	Nimodipine	-35.673912
DB01102	Arbutamine	-35.671391
DB01023	Felodipine	-35.645214
DB06243	Eflornithine	-35.634377
DB12127	Sultamicillin	-35.629509
DB00580	Valdecoxib	-35.628311
DB01587	Ketazolam	-35.623104
DB06261	Hexaminolevulinate	-35.617867
DB08884	Gadoxetic acid	-35.614735
DB09241	Methylene blue	-35.610668
DB12710	Perazine	-35.597878
DB11129	Carbamide peroxide	-35.594818
DB11748	Benfotiamine	-35.58292
DB01260	Desonide	-35.579426
DB09084	Benzydamine	-35.579388
DB03247	Flavin mononucleotide	-35.578377
DB08967	Dimetotiazine	-35.575943
DB09030	Vorapaxar	-35.538311
DB01056	Tocainide	-35.536057
DB06791	Lanreotide	-35.52824
DB09086	Eugenol	-35.51569
DB00551	Acetohydroxamic acid	-35.510853
DB00336	Nitrofural	-35.494518
DB08976	Floctafenine	-35.487545
DB14642	Lypressin	-35.483704

DB00990	Exemestane	-35.473175
DB00245	Benzatropine	-35.467636
DB00142	Glutamic acid	-35.463757
DB02513	Thymol	-35.44891
DB01249	Iodixanol	-35.439289
DB13221	Apronalide	-35.433754
DB01297	Practolol	-35.353531
DB12404	Remimazolam	-35.3526
DB06267	Udenafil	-35.333168
DB08864	Rilpivirine	-35.330936
DB01132	Pioglitazone	-35.309719
DB00635	Prednisone	-35.304569
DB00534	Chlormerodrin	-35.285721
DB00920	Ketotifen	-35.282619
DB12130	Lorlatinib	-35.282585
DB00592	Piperazine	-35.278297
DB00838	Clocortolone	-35.261089
DB01261	Sitagliptin	-35.252094
DB09220	Nicorandil	-35.188869
DB08985	Etilefrine	-35.184494
DB12362	Diaminopropanol tetraacetic acid	-35.173962
DB00946	Phenprocoumon	-35.120941
DB09120	Zucapsaicin	-35.104301
DB13800	Calcium levulinate	-35.089542
DB12783	Benserazide	-35.084396
DB01123	Proflavine	-35.065399
DB00988	Dopamine	-35.063828
DB11952	Duvelisib	-35.054108
DB00678	Losartan	-35.038563
DB12537	Benzodiazepine	-35.029713
DB01436	Alfacalcidol	-35.006516
DB08911	Trametinib	-35.001152
DB09123	Dienogest	-34.985195
DB01407	Clenbuterol	-34.971458
DB06817	Raltegravir	-34.954967
DB06282	Levocetirizine	-34.930077
DB00310	Chlorthalidone	-34.918991
DB00199	Erythromycin	-34.901123
DB01086	Benzocaine	-34.898739
DB09065	Cobicistat	-34.887077
DB08818	Hyaluronic acid	-34.873566
DB09143	Sonidegib	-34.862404
DB09124	Medrogestone	-34.844917
DB01601	Lopinavir	-34.83511

DB09382	Iodohippurate sodium I-131	-34.820267
DB06210	Eltrombopag	-34.793999
DB00444	Teniposide	-34.791267
DB09269	Phenylacetic acid	-34.774979
DB00378	Dydrogesterone	-34.758862
DB00641	Simvastatin	-34.734776
DB14201	2 2'-Dibenzothiazyl disulfide	-34.706059
DB01330	Cefotetan	-34.702072
DB00977	Ethinylestradiol	-34.687618
DB11735	Galactose	-34.651745
DB11191	Cobamamide	-34.609241
DB00480	Lenalidomide	-34.593704
DB00776	Oxcarbazepine	-34.569855
DB01188	Ciclopirox	-34.567131
DB00406	Gentian violet cation	-34.559246
DB09389	Norgestrel	-34.5424
DB08809	Dichloroacetic acid	-34.533165
DB00593	Ethosuximide	-34.528183
DB14656	Chlorphenesin carbamate	-34.528095
DB00911	Tinidazole	-34.50993
DB00204	Dofetilide	-34.49538
DB04813	Bithionol	-34.492592
DB00357	Aminoglutethimide	-34.488239
DB00726	Trimipramine	-34.485718
DB11943	Delafloxacin	-34.476627
DB04896	Milnacipran	-34.473892
DB14646	Prednisone acetate	-34.456375
DB00856	Chlorphenesin	-34.443863
DB11583	Cetalkonium	-34.414219
DB09068	Vortioxetine	-34.413822
DB01122	Ambenonium	-34.378193
DB09324	Sulbactam	-34.3643
DB00552	Pentostatin	-34.363956
DB14529	Chromium nicotinate	-34.361832
DB00284	Acarbose	-34.342056
DB01064	Isoprenaline	-34.337597
DB00411	Carbamoylcholine	-34.33757
DB09374	Indocyanine green acid form	-34.336361
DB01607	Ticarcillin	-34.329941
DB00899	Remifentanil	-34.329922
DB00722	Lisinopril	-34.326733
DB00209	Trospium	-34.314137
DB14203	Disperse Blue 106	-34.305302
DB00433	Prochlorperazine	-34.303501

DB00892	Oxybuprocaine	-34.293987
DB12978	Pexidartinib	-34.290955
DB14568	Ivosidenib	-34.276604
DB05057	Erdosteine	-34.276203
DB14575	Eslicarbazepine	-34.248127
DB01214	Metipranolol	-34.242313
DB00830	Phenmetrazine	-34.238136
DB00350	Minoxidil	-34.233761
DB01262	Decitabine	-34.199131
DB01218	Halofantrine	-34.179764
DB00907	Cocaine	-34.172596
DB09300	Butylscopolamine	-34.160442
DB00683	Midazolam	-34.123356
DB14505	Sodium borate	-34.108776
DB00132	alpha-Linolenic acid	-34.092918
DB00669	Sumatriptan	-34.05788
DB09063	Ceritinib	-34.049709
DB00194	Vidarabine	-34.047066
DB00607	Nafcillin	-34.045986
DB01176	Cyclizine	-34.025219
DB00280	Disopyramide	-34.023685
DB11730	Ribociclib	-34.017845
DB00596	Ulobetasol	-34.007004
DB09333	Iopodic acid	-33.987663
DB00835	Brompheniramine	-33.980297
DB00869	Dorzolamide	-33.934837
DB13170	Plecanatide	-33.931461
DB00915	Amantadine	-33.918987
DB00243	Ranolazine	-33.910572
DB12035	Sarecycline	-33.908916
DB01583	Liotrix	-33.890026
DB12015	Alpelisib	-33.888615
DB00450	Droperidol	-33.888039
DB01008	Busulfan	-33.884167
DB00573	Fenoprofen	-33.880989
DB01149	Nefazodone	-33.871014
DB13292	Pimethixene	-33.866207
DB00731	Nateglinide	-33.862564
DB00421	Spironolactone	-33.855373
DB06804	Nonoxynol-9	-33.834763
DB00297	Bupivacaine	-33.832714
DB00173	Adenine	-33.818249
DB06287	Temsirolimus	-33.811008
DB00885	Pemirolast	-33.798351

DB00933	Mesoridazine	-33.798302
DB06441	Cangrelor	-33.797424
DB09079	Nintedanib	-33.793911
DB14212	Methylparaben	-33.792564
DB11921	Deflazacort	-33.745628
DB11328	Tetradecyl hydrogen sulfate (ester)	-33.743778
DB00140	Riboflavin	-33.696465
DB04839	Cyproterone acetate	-33.696259
DB01421	Paromomycin	-33.695461
DB00367	Levonorgestrel	-33.683315
DB00445	Epirubicin	-33.674339
DB06414	Etravirine	-33.669144
DB00880	Chlorothiazide	-33.660923
DB13874	Enasidenib	-33.648621
DB01029	Irbesartan	-33.635418
DB01140	Cefadroxil	-33.625004
DB01616	Alverine	-33.62426
DB01193	Acebutolol	-33.62299
DB04837	Clofedanol	-33.599339
DB01155	Gemifloxacin	-33.595589
DB12243	Edaravone	-33.593693
DB00798	Gentamicin	-33.584194
DB06147	Sulfathiazole	-33.581722
DB01197	Captopril	-33.570518
DB00206	Reserpine	-33.56881
DB11183	Light green SF yellowish	-33.553955
DB12161	Deutetrabenazine	-33.484615
DB00789	Gadopentetic acid	-33.478409
DB01141	Micafungin	-33.469616
DB14541	Hydrocortisone cypionate	-33.454403
DB00502	Haloperidol	-33.449436
DB01299	Sulfadoxine	-33.440716
DB05351	Dexlansoprazole	-33.425941
DB03615	Ribostamycin	-33.410297
DB00458	Imipramine	-33.403492
DB00397	Phenylpropanolamine	-33.400757
DB11581	Venetoclax	-33.374088
DB00978	Lomefloxacin	-33.358459
DB14723	Larotrectinib	-33.357994
DB11264	Calcium glycerophosphate	-33.340721
DB11611	Lifitegrast	-33.33633
DB00650	Leucovorin	-33.324837
DB06590	Ceftaroline fosamil	-33.318302
DB01576	Dextroamphetamine	-33.316048

DB00389	Carbimazole	-33.305573
DB06766	Alcaftadine	-33.300789
DB01399	Salsalate	-33.290783
DB01239	Chlorprothixene	-33.264046
DB01131	Proguanil	-33.256451
DB01325	Quinethazone	-33.250141
DB00451	Levothyroxine	-33.234818
DB01095	Fluvastatin	-33.233345
DB00376	Trihexyphenidyl	-33.214664
DB11100	Allantoin	-33.180225
DB00192	Indecainide	-33.177418
DB00693	Fluorescein	-33.176262
DB11304	Phenoxyethanol	-33.168709
DB00555	Lamotrigine	-33.165859
DB01007	Tioconazole	-33.156857
DB04843	Mepenzolate	-33.15506
DB00662	Trimethobenzamide	-33.142956
DB09297	Paritaprevir	-33.136436
DB00843	Donepezil	-33.117889
DB00825	Levomenthol	-33.111012
DB01605	Pivmecillinam	-33.110535
DB00420	Promazine	-33.109249
DB11796	Fostemsavir	-33.075474
DB00700	Eplerenone	-33.075123
DB00150	Tryptophan	-33.056458
DB01783	Pantothenic acid	-33.037563
DB00684	Tobramycin	-33.032856
DB00807	Proparacaine	-33.032646
DB00530	Erlotinib	-33.027428
DB13245	Thiram	-33.015423
DB01224	Quetiapine	-33.012741
DB01015	Sulfamethoxazole	-33.007355
DB00477	Chlorpromazine	-33.001667
DB08828	Vismodegib	-32.984837
DB11963	Dacomitinib	-32.983524
DB00782	Propantheline	-32.966343
DB11689	Selumetinib	-32.945511
DB06711	Naphazoline	-32.908455
DB09488	Acrivastine	-32.903278
DB00273	Topiramate	-32.891026
DB00973	Ezetimibe	-32.886623
DB00904	Ondansetron	-32.856716
DB01121	Phenacemide	-32.853748
DB00640	Adenosine	-32.848209

DB01181	Ifosfamide	-32.842243
DB00275	Olmesartan	-32.828117
DB00828	Fosfomycin	-32.824032
DB01394	Colchicine	-32.814198
DB01096	Oxamniquine	-32.764385
DB04834	Rapacuronium	-32.743382
DB00368	Norepinephrine	-32.734615
DB06202	Lasofoxifene	-32.706669
DB00481	Raloxifene	-32.67617
DB05316	Pimavanserin	-32.662868
DB14198	Quaternium-15	-32.652512
DB00364	Sucralfate	-32.645039
DB04794	Bifonazole	-32.638592
DB14476	DL-alpha-Tocopherol	-32.634834
DB09397	Technetium Tc-99m sulfur colloid	-32.620174
DB00608	Chloroquine	-32.589603
DB01603	Meticillin	-32.582211
DB13879	Glecaprevir	-32.554245
DB11584	Pipradrol	-32.545578
DB00763	Methimazole	-32.543907
DB01110	Miconazole	-32.522663
DB14490	Ferrous ascorbate	-32.521561
DB06603	Panobinostat	-32.51926
DB00563	Methotrexate	-32.51825
DB09296	Ombitasvir	-32.503033
DB11121	Chloroxylenol	-32.495331
DB04898	Ximelagatran	-32.49379
DB00443	Betamethasone	-32.450878
DB00603	Medroxyprogesterone acetate	-32.444332
DB01143	Amifostine	-32.419834
DB00576	Sulfamethizole	-32.412491
DB00829	Diazepam	-32.409393
DB00572	Atropine	-32.396397
DB00471	Montelukast	-32.373009
DB14753	Hydroxystilbamidine	-32.372322
DB09094	Podophyllin	-32.370953
DB00163	Vitamin E	-32.368343
DB04871	Lorcaserin	-32.362061
DB00430	Cefpiramide	-32.347519
DB00804	Dicyclomine	-32.343681
DB13960	Bronopol	-32.34214
DB06702	Fesoterodine	-32.341602
DB06402	Telavancin	-32.338245
DB00827	Cinoxacin	-32.324493

DB01120	Gliclazide	-32.319489
DB13854	Gamolenic acid	-32.309711
DB14199	Bromothalonil	-32.287239
DB08909	Glycerol phenylbutyrate	-32.285877
DB09038	Empagliflozin	-32.273003
DB11817	Baricitinib	-32.256805
DB00964	Apraclonidine	-32.250679
DB11323	Glycol salicylate	-32.249252
DB00403	Ceruletide	-32.227829
DB00967	Desloratadine	-32.224121
DB11644	Tafamidis	-32.211773
DB05381	Histamine	-32.211372
DB00396	Progesterone	-32.194332
DB13273	Sultopride	-32.186569
DB00706	Tamsulosin	-32.179497
DB01230	Pemoline	-32.1758
DB14491	Ferrous fumarate	-32.166206
DB00207	Azithromycin	-32.154335
DB09212	Loxoprofen	-32.138233
DB00754	Ethotoin	-32.136368
DB01221	Ketamine	-32.132629
DB01353	Butobarbital	-32.127758
DB09031	Miltefosine	-32.125137
DB12615	Plazomicin	-32.121525
DB00390	Digoxin	-32.120476
DB06755	Beta carotene	-32.115917
DB01366	Procaterol	-32.112049
DB08941	Isoxsuprine	-32.108341
DB01364	Ephedrine	-32.103718
DB01072	Atazanavir	-32.084248
DB11678	Treosulfan	-32.077557
DB01078	Deslanoside	-32.0555
DB00703	Methazolamide	-32.055191
DB01192	Oxymorphone	-32.053501
DB00422	Methylphenidate	-32.053097
DB00759	Tetracycline	-32.039097
DB00400	Griseofulvin	-32.035343
DB01625	Isopropamide	-32.03331
DB06616	Bosutinib	-32.028645
DB04743	Nimesulide	-31.996775
DB08819	Tafluprost	-31.98056
DB00295	Morphine	-31.97196
DB06797	Mebutamate	-31.922071
DB12834	Secnidazole	-31.911392

DB00602	Ivermectin	-31.90851
DB11677	Triheptanoin	-31.90279
DB09117	Paraldehyde	-31.899319
DB05016	Ataluren	-31.898621
DB00441	Gemcitabine	-31.889408
DB04209	Dequalinium	-31.881844
DB01190	Clindamycin	-31.874359
DB00742	Mannitol	-31.850048
DB08816	Ticagrelor	-31.845417
DB00370	Mirtazapine	-31.84269
DB09071	Tasimelteon	-31.832113
DB06821	Sulfameter	-31.826385
DB00266	Dicoumarol	-31.810425
DB12153	Citicoline	-31.803337
DB09291	Rolapitant	-31.782711
DB00996	Gabapentin	-31.782393
DB00230	Pregabalin	-31.78236
DB11823	Esketamine	-31.764196
DB00801	Halazepam	-31.742079
DB00980	Ramelteon	-31.722422
DB02638	Terlipressin	-31.716263
DB01045	Rifampicin	-31.704542
DB00287	Travoprost	-31.702534
DB00577	Valaciclovir	-31.683874
DB01051	Novobiocin	-31.665489
DB00419	Miglustat	-31.656992
DB14540	Hydrocortisone butyrate	-31.638771
DB01156	Bupropion	-31.629129
DB01133	Tiludronic acid	-31.626888
DB11855	Revefenacin	-31.624126
DB08995	Diosmin	-31.611881
DB04221	Didecyldimethylammonium	-31.608194
DB00741	Hydrocortisone	-31.600113
DB00239	Oxiconazole	-31.59807
DB14176	Benzylparaben	-31.592373
